# Mediators and moderators of the effects of the COVID-19 crisis on parent–child conflict in children in tertiary mental health care

**DOI:** 10.1038/s41598-023-49409-2

**Published:** 2023-12-16

**Authors:** Philippe Robaey, Madison Erbach, Lori K. Watanabe, Elizabeth R. Carreiro, Alexander R. Maisonneuve

**Affiliations:** 1 Children’s Hospital of Eastern Ontario, Research Institute, 401 Smyth Road, Ottawa, ON K1H8L1 Canada; 2 Department of Psychiatry, University of Ottawa, Ottawa, ON Canada; 3 Interdisciplinary School of Health Sciences, University of Ottawa, Ottawa, ON Canada

**Keywords:** Psychology, Paediatrics

## Abstract

This study focused on children treated for mental health problems during the pandemic. The present study examined how parent’s difficulties in managing COVID-19 restrictions increased children's behavioral problems (internalizing and externalizing) and parent–child conflict through parental mental health and parental stress. Family functioning, particularly problem-solving ability, was tested as a resilience factor. were collected using online surveys from 337 parents with a child between the ages of 4 and 18 years who was receiving active outpatient mental health treatment at a pediatric tertiary care center. Parents who reported a greater impact of COVID-19 reported more behavioral difficulties in their children. This relationship was significantly mediated by parental mental health (general stress, anxiety, and depression) and parental stress. Similar indirect pathways were observed when examining internalizing and externalizing problems in children, where the most significant pathway had parental stress as the sole mediator. Furthermore, the effect of COVID-19 impact on parent–child conflict through parental stress was significantly moderated by problem-solving skills within the family. Parenting stress mediates the impact of COVID-19 on parent–child conflict. Interventions improving within family problem solving-skills may decrease the effect of parental stress on parent–child conflict.

## Introduction

Since the onset of the pandemic various studies have explored in the general population the deterioration of the mental health of children during the COVID-19 crisis especially by using a Family Stress Model^1,2^. However, to our knowledge, these mechanisms have yet to be investigated in a sample of children followed for mental health problems during the pandemic. This study aims to fill this research gap.

The World Health Organization declared COVID-19 as a pandemic on March 11, 2020. A few days later, on March 17, the premier of Ontario declared a state of emergency in the province. In the subsequent months, Ontario experienced repeated “lockdowns” with partial to complete closures of workplaces, schools, parks, childcare, and non-essential businesses. From March 14, 2020, to May 15, 2021, Ontario schools (including special education classes and day care centers) had been closed for 20 weeks. In the education system, remote learning was quickly implemented. In the health care system, particularly as it relates to mental health, face-to-face visits were rapidly replaced by virtual visits, except for some emergencies.

The immediate impact of COVID-19 on the mental health of children and youth in the general population was soon documented. For example, in Ontario, more than two-thirds of children and youth experienced a deterioration in their mental health, regardless of age. Greater stress was associated with deterioration in all areas of mental health^3^. These findings have been replicated worldwide. A meta-analysis confirmed that confinement measures were associated with worsening mental health in children but the effect was small. Effect sizes were larger in European countries than in Asian countries^4^.

Other studies have aimed to understand the direct and indirect mechanisms affecting children's mental health. In the aftermath of a disaster, children's mental health is not only affected by direct exposure to its direct victims or effects, but also by its indirect impact on parental stress, parenting practices and parent–child interactions, as shown in the following studies. After Hurricane Katrina, some causal pathways to PTSD were through parental distress and parenting practices^5^. Compared to such immediate disasters (such as floods, fires, accidents, and wars), the COVID-19 crisis is unique in that its immediate cause, a virus, is invisible, in that most children had no direct experience with the disease and its victims. Instead, they were exposed to profound disruptions to their daily routines as schools, recreational facilities, non-essential businesses closed, and health care systems were significantly impacted. The COVID-19 crisis increased parents' general stress, but also more specifically their parental stress, by imposing additional, unprecedented demands on them, different from those they expected before the crisis (e.g. isolation, distance learning, explanations of measures), and for which their coping resources may be insufficient^6^. To take one example, school closures and distance learning contributed to increased levels of parental stress even after controlling for stressful life events (accidents, moves, etc.) and stress directly related to COVID-19 itself (such as the fear of contracting COVID-19)^7^.

Parental stress in turn affected children’s mental health during the COVID-19 crisis. This has been found in the general population in different cultures. In Singapore, parental stress mediated the effect of the perceived impact of COVID-19 on harsh parenting and decreased parent–child closeness^8^. In Italy, parents who reported more difficulties in dealing with quarantine showed more parental stress, which in turn affected parental reports of conduct problems, emotional symptoms, and hyperactivity/inattention in their children^9^. Although these previous studies often did not explicitly refer to it, they used the family stress model (FSM) first proposed by Conger in 1994^1^.

The FSM suggests that financial difficulties increase parents' stress, which affects their parenting, which in turn increases the risk of academic, psychological, and behavioral problems in their children. These studies replaced poverty by COVID-19 in the same model. The FSM then evolved by proposing new mediating pathways, and factors that moderate mediating pathways^2^. Following these developments protective pathways have been explored. While parental stress emerged as a risk factor for children’s mental health, parental sense of competency, and some specific parenting practices, emerged as protective factors in COVID-19 pandemic studies, as shown in the following two studies. Parental stress related to distance learning was reduced in parents with high perceived parental self-efficacy^7^. While parental distress had a negative impact on children's mental health, ensuring structure and regularity, emotional comfort, or good communication were linked to positive outcomes^10^. Beyond parenting skills and feelings of competence, protective factors in family functioning must also be considered. Resilience processes have been identified in three areas of family functioning: family belief systems, organizational patterns, and communication/problem-solving skills^11^. We selected the latter as a moderator of the parenting stress pathway to child mental health, and thus also as a potential target for interventions.

If the mediating role of parental stress in the pathway from the COVID-19 disruption of daily routine to children’s mental health outcomes seems well established, it has only been studied in the general population. It is not immediately clear that parental stress plays the same role with children currently being treated for mental health problems. Parents of these children were already experiencing high parental stress before the pandemic^12,13^, so the impact of the COVID-19 crisis might be less severe than in the general population because of a ceiling effect. In addition, some aspects of parenting stress, such as those related to school avoidance or peer conflicts, may be attenuated when children were not attending school in person, and had much less interactions with peers^14^.

In this context, we designed a step-by-step approach for analyzing the data. Our primary hypothesis was that parental stress was the main mediator of the effect of COVID-19, and parent–child conflict the main outcome. But first, we wanted to differentiate between fear and the impact of COVID-19 and compare the effect of both aspects on pathways to child mental health outcomes. Second, as parents of children with mental health problems often report mental health problems themselves (which proved true in this sample), we also introduced the intermediate mediators of general stress, anxiety and depression leading to parental stress into the models, in order to compare single-stage and multi-stage pathways. Third, we compared the pathways leading to different child mental health outcomes: general, internalized disorders, externalized disorders, and parent child conflict to identify which outcome is most affected by the pathway from COVID-19 through parental stress. We compared the 15 possible pathways between the two aspects of COVID-19 and the different child mental health outcomes using standardized coefficients. Finally, with the most informative model, we tested whether the mediator's effect on outcome was moderated by family conflict resolution competence, which could potentially be enhanced by intervention, and mitigate the effects of COVID-19.

Therefore, we recruited families with children treated at an outpatient mental health clinic during the first year of the COVID-19 crisis. We assessed the effects of fear of COVID-19 and also the difficulty in dealing with the measures implemented during the COVID-19 crisis. As possible mediators, we assessed parental mental health and parental stress levels. Child mental health problems and parent–child conflict were the primary outcomes. As moderator, we assessed family functioning.

## Methods

### Participants

Parents were eligible if they had a child aged 4 to 18 years old who had an active clinical health record in the outpatient mental health clinic of the Children’s Hospital of Eastern Ontario (CHEO). To have an active file, patients must be referred by their family physician to CHEO, which is the only pediatric tertiary care center in the Ottawa region. In general, mental health problems must be serious and resistant to treatment in the community for referrals to be accepted. There were no exclusion criteria other than age. Approximately one third of the children were between 4 and 12 years old, the second third were between 12 and 15 years old, and the last third were between 15 and 18 years old. Respondents were primarily biological mothers (*n* = 270, 80.1%) followed by fathers (*n* = 61, 18.1%) and other guardians (*n* = 5, 1.5%).

### Procedure

**Figure 1:**
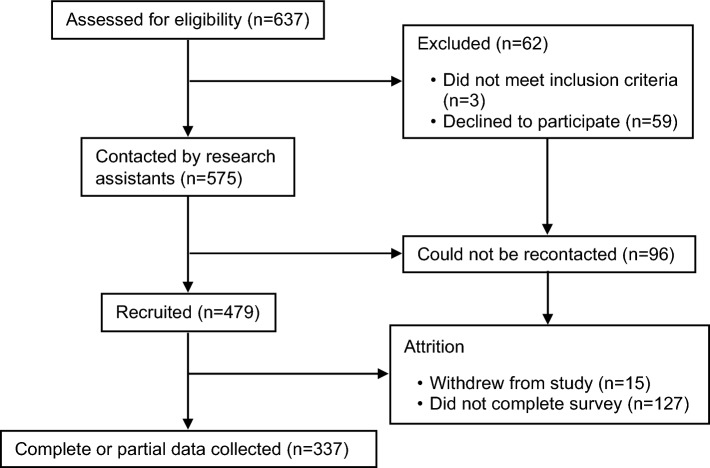
Participant flow diagram.

See Fig. 1 for a participant flow diagram. Eligible families were identified using their child’s electronic medical record. This study was approved by the Children’s Hospital of Eastern Ontario Research Ethics Board (REB). In accordance with REB rules, hospital staff initially contacted 637 parents by telephone to determine if they were willing to be contacted by the research team. A research assistant recontacted the parents who had consented to confirm their participation. Using this procedure, 479 participants were recruited between May 2020 and May 2021. Participants completed an electronic informed consent form and then completed a general demographic questionnaire that documented their health, financial, housing, and family status. Participants received a unique link to a series of questionnaires (see below). Complete and partial data was collected from 337 participants (*Mean age* = 45.90 years, *SD* = 6.84 years). Participants who completed only partially the survey were not different from those that fully completed it in terms of age, COVID fear and impact, PSS parental stress, DASS stress, anxiety and depression and FAS problem solving (see Appendix A). The other 127 participants did not respond to the questionnaires (i.e., only demographic information was collected).

### Measures

#### Fear of COVID-19

The “Fear of COVID-19 Scale”^15^ is a 7-item scale that measures fears, worries, and anxieties relating to COVID-19. Each item is scored using a 5-point Likert scale ranging from “strongly disagree” to “strongly agree”. Item scores are summed; higher scores indicate a greater fear of COVID-19. Reliability values such as internal consistency (α = 0.82) and test–retest reliability (ICC = 0.72) were acceptable.

#### Impact of COVID-19

The impact of COVID-19 was assessed using 22 newly created items to assess parents’ difficulties with confinement, isolation, technology/internet, remote schoolwork, home chores, childcare, family life, and COVID-19 rules. Items were scored on a 5-point Likert scale ranging from “strongly disagree” to “strongly agree”. The objective was to create a scale to assess difficulty in dealing with the measures put in place during the COVID-19 crisis that was as independent as possible from the fear of COVID-19, so that their specific effects could be evaluated separately. We first ran an exploratory factor analysis combining the two scales, which produced an Impact and a Fear factor. All but two of the newly created Impact items loaded on the Impact factor, and these two items were deleted. Seven items with a loading less than 0.4 on the Impact factor were also deleted, which resulted in a 13-item Impact Scale. Following that, we ran a factorial analysis on the 20 Fear and Impact items using generalized least squares as an extraction method and limiting the number of factors to two. A two-factor solution was supported (χ^2^(229) = 514.8; *p* < 0.0001). Factor analysis demonstrated good internal consistency (α = 0.843) for the Impact items. The total COVID-19 Impact score is calculated by summing the 13 items, with higher scores indicating greater challenges with the COVID-19 crisis. The retained items on the COVID-19 Impact Scale assessed difficulties dealing with the lockdown (change in leisure, chores, home-schooling), but also difficulties with childcare and online access (Appendix B). The correlation between the Impact and Fear scales was moderate, but still significant: *r*(311) = 0.31, *p* < 0.001.

#### Parent mental health

Parent mental health was assessed using the Depression Anxiety Stress Scales (DASS^16^). The DASS is a 42-item measure with three subscales that assess self-reported depression, anxiety, and stress. The DASS has good internal consistency (Depression α = 0.91; Anxiety 0.84; Stress 0.90). The DASS Anxiety scale correlated 0.81 with the Beck Anxiety Inventory, and the DASS Depression scale correlated 0.74 with the Beck Depression Inventory^16,17^. Items are rated on a 4-point Likert scale. Item scores are then summed for each subscale, with higher scores indicating greater self-reported depression, anxiety, or stress. Raw subscale scores were converted to percentiles using established norms^17^.

#### Parental stress

Parental stress was measured using the 18-item Parental Stress Scale (PSS^18^). Each item is rated on a 5-point Likert scale. Higher total scores range from 18 to 90 with higher scores indicating greater levels of parenting stress. The PSS has good internal consistency (α = 0.801) and convergent validity with the Parenting Stress Index total score (r = 0.75)^18^.

#### Family functioning

The Family Assessment Device (FAD^19^) is a 60-items self-report instrument developed to assess seven dimensions relating to family functioning and the overall pathology of a family. Items are scored on a 4-point Likert scale. In this study, the 6-item Problem Solving subscale was used. The Problem Solving subscale measures a family’s ability to resolve problems and maintain effective family functioning, with higher scores indicating lower problem-solving skills. The Problem Solving subscale has good internal consistency (α = 0.74).

#### Child behavioural difficulties

The Strengths and Difficulties Questionnaire (SDQ^20,21^) is a 25-item questionnaire in which parents report on the positive and negative attributes of their child. Each item is rated on a 3-point Likert scale. Four of the subscales (Emotional Symptoms, Conduct Problems, Hyperactivity/Inattention, and Peer Relationship Problems) are combined to form a total difficulties score. In addition to using the total difficulties score as an outcome, we also investigated internalizing (Emotional Symptoms and Peer Relationship) and externalizing (Conduct Problems and Hyperactivity/Inattention) problems. Age and gender norms from a sample of American children^22^ were used to translate raw scores into percentiles. Internal consistency (mean Cronbach α: 0.73) and retest stability after 4 to 6 months (mean: 0.62) were satisfactory^23^. The time covered by SDQ is the last 6 months. In order to focus on the lockdown period, participants were instructed to rate the behavior especially during the COVID-19 situation.

#### Parent–child conflict

Parent–child conflict was assessed using the 90-item Family Environment Scale (PEQ^24,25^). In this study, only the 12-item Parent–Child Conflict subscale was administered. Internal consistency (alpha = − 0.88) suggests that conflict is a consistently reliable construct^26^. Each item is answered on a 4-point Likert scale with higher scores indicating higher parent–child conflict. The 12 items cover various aspects of the parent–child conflict asking the parent what they do (e.g., I often irritate my child, I often criticize my child, etc.) but also what the child does (e.g., My child often angers or annoys me, My child has been really scared of me, etc.) or what both do (e.g., My child and I often argue, I often have misunderstandings with my child).

#### Demographic information

Information about the participant’s health (COVID-19 status, acute and chronic physical health conditions and mental health conditions), work (working from home, job loss due to COVID-19, essential worker), housing (type of home, type of community), and family status (marital status, custody arrangement) was collected. Three items assessed the participant’s financial status: pre-pandemic income, percentage decrease in income during the pandemic, and additional sources of income received (e.g., none, Canada Emergency Response Benefit, disability support).

### Analytic plan

In terms of missing data, subscale scores for the SDQ and the FAD were calculated with the available data when < 40% of the items were missing for a given subscale. For all other measures, missing values were replaced with the series mean when < 40% of data was missing. Missing data were not replaced when ≥ 40% of data was missing.

Data were analyzed using SPSS version 28. We performed mediation analyses using the SPSS macro PROCESS v4.1^27^. First, we tested a mediation model with the DASS stress, anxiety, and depression variables, in addition to parental stress (model 6). All analyses included the following variables as covariates: child’s age, child’s gender, number of days elapsed since emergency state declaration, reported income level, and percentage decrease in income since the start of the pandemic. In addition, we used the COVID-19 Fear scores as covariables when testing the effect of COVID-19 Impact scores, and vice-versa, as both variables were correlated. As a second step, we retained only the most important mediators and tested the moderating effect of family functioning variables on the direct and indirect pathways of COVID-19 impact on mental health outcome (model 88). The purpose of this analysis was to test whether certain characteristics of family functioning could moderate the indirect effects of the mediators, and thus be a target for an intervention to decrease the impact of COVID-19 on children’s mental health.

## Results

### Descriptive statistics and correlations (Tables 1, 2 and 3)

**Table 1:** Sample characteristics for n = 337.

Variable	N	%	M	SD	Range
Days elapsed			183.72	71.70	78–401
Child age			13.30	3.43	4.2–18.8
Child gender					
Male	189	56.1			
Female	147	43.6			
Marital status					
Married/common law	219	65.0			
Divorced/separated	82	24.3			
Single	28	8.3			
Widowed	7	2.1			
Income					
< $50,000	49	14.5			
$50,000–$99,999	90	27.6			
$100,000–$149,999	65	19.3			
$150,000–$199,999	42	12.5			
> $200,000	40	11.9			
Missing	51	15.1			
Income decrease					
No change (0%)	218	64.7			
10–30% decrease	63	18.7			
40–60% decrease	22	6.5			
70–100% decrease	9	2.7			
Missing	25	7.4			
Type of community					
Urban	104	30.9			
Suburban	173	51.3			
Rural	57	16.9			
Custody					
Parental	219	65.0			
Sole	75	22.2			
Joint	40	11.9			
Crown ward	2	0.6			
Parent health condition					
Chronic physical	115	34.1			
Acute physical	23	6.8			
COVID-19	9	2.7			
Mental health	140	41.5			

Percentages in the table do not include missing data and may not total 100%.

**Table 2:** Descriptive statistics of study variables for N = 337.

Variable	N	Raw scores			Percentiles		
		M	SD	Range	M	SD	Range
COVID impact	330	54.45	11.98	7–87			
COVID fear	332	17.40	5.59	7–34			
DASS stress	259	14.02	9.76	0–42	73.24	28.65	5–99
DASS anxiety	259	6.46	7.72	0–42	74.82	24.66	30–99
DASS depression	259	10.30	10.11	0–42	75.39	25.47	20–99
PSS parental stress	251	46.41	11.30	18–85			
SDQ total	259	19.90	6.92	1–38			
SDQ internalizing	258				89.04	12.30	38–99
SDQ externalizing	258				85.52	14.27	35–99

DASS: Depression Anxiety Stress Scales, PSS: Parental Stress Scale, SDQ: Strengths and Difficulties Questionnaire.

**Table 3:** Correlations between main study variables and covariates.

	Variable	1	2	3	4	5	6	7	8	9	10	11	12	13
1.	COVID fear	–												
2.	COVID impact	0.31***	–											
3.	DASS stress	0.31***	0.40***	–										
4.	DASS anxiety	0.34***	0.30***	0.74***	–									
5.	DASS depression	0.21***	0.37***	0.78***	0.72***	–								
6.	PSS parental stress	0.12*	0.51***	0.49***	0.32***	0.53***	–							
7.	SDQ total child difficulties	0.12*	0.31***	0.26***	0.21***	0.21***	0.47***	–						
8.	SDQ internalizing	0.10	0.18**	0.13*	0.15*	0.13*	0.28***	0.66***	–					
9.	SDQ externalizing	0.12	0.31***	0.27***	0.16*	0.21***	0.44***	0.79***	0.34***	–				
10.	PEQ parent–child conflict	0.07	0.33***	0.45***	0.21***	0.36***	0.61***	0.53***	0.25***	0.55***	–			
11.	Child age	− 0.13*	− 0.14*	− 0.09	− 0.07	− 0.05	− 0.01	− 0.07	0.09	− 0.09	0.09	–		
12.	Days elapsed	0.15*	0.03	0.15*	0.14*	0.01	0.12	0.03	0.07	− 0.04	0.08	–0.06	–	
13.	Income	− 0.16**	− 0.12*	− 0.04	− 0.22***	− 0.16*	< 0.01	− 0.04	− 0.06	< 0.01	0.05	0.10	–0.12	–
14.	Income % decrease	0.13*	0.01	0.01	0.07	0.07	0.03	0.06	0.05	0.11	− 0.03	− 0.02	− 0.04	− 0.13*

*p < 0.05, **p < 0.01, ***p < 0.001.

Reflecting the clinical nature of the sample, about two third of the children (68.3%) were reported as having a total SDQ score in the high (17–19 range) or very high range (20–40 range). When considering the SDQ subscales separately, the percentage of children in the high/very high range was 64.9% for the emotional subscale, 45.1% for the conduct problem, 44% for hyperactivity, 62.5% for the peer problem, and 53% for low or very low prosocial subscales. The full distribution of scores is presented in Appendix C (in order to further assess the functioning of the patients, we retrieved from the health record the scores entered by clinicians for the Children's Global Assessment Scale (CGAS)^28^. Only 56% of the patients had at least 1 CGAS from March 2020 to May 2021. Only 4% of the mean CGAS were in the normal functional range (> 70). Their total SDQ was 4.7 points lower than those with no CGAS. The correlation between CGAS and SDQ total was low but significant (r − 0.164; p < 0.05). Although the reliability of these CGAS scores is questionable, they reflect the severe limitation in functioning of this sample.)

For the parents, DASS showed high average levels of depresssion (75th percentile), anxiety (75th percentile), and stress (73th percentile) scores. The first third of the questionnaires were completed within the first 150 days of the pandemic, the second third in the following 50 days and the remainder up to day 401. Approximately one-third of the sample was between 4 and 12 years of age, the second third between 12 and 15 years, and the final third between 15 and 18 years.

Greater COVID-19 impact was significantly associated with lower income and younger age (ps < 0.05), higher internalizing (p < 0.01), and higher externalizing problems in children, more parent–child conflict, as well as higher stress, anxiety, depression, and parental stress in parents (ps < 0.001). Lower income was associated with higher anxiety (p < 0.001), depression (p < 0.05), impact (p < 0.01) and fear (p < 0.05) of COVID-19 scores in parents.

### Mediation analyses

#### SDQ total scores

**Figure 2:**
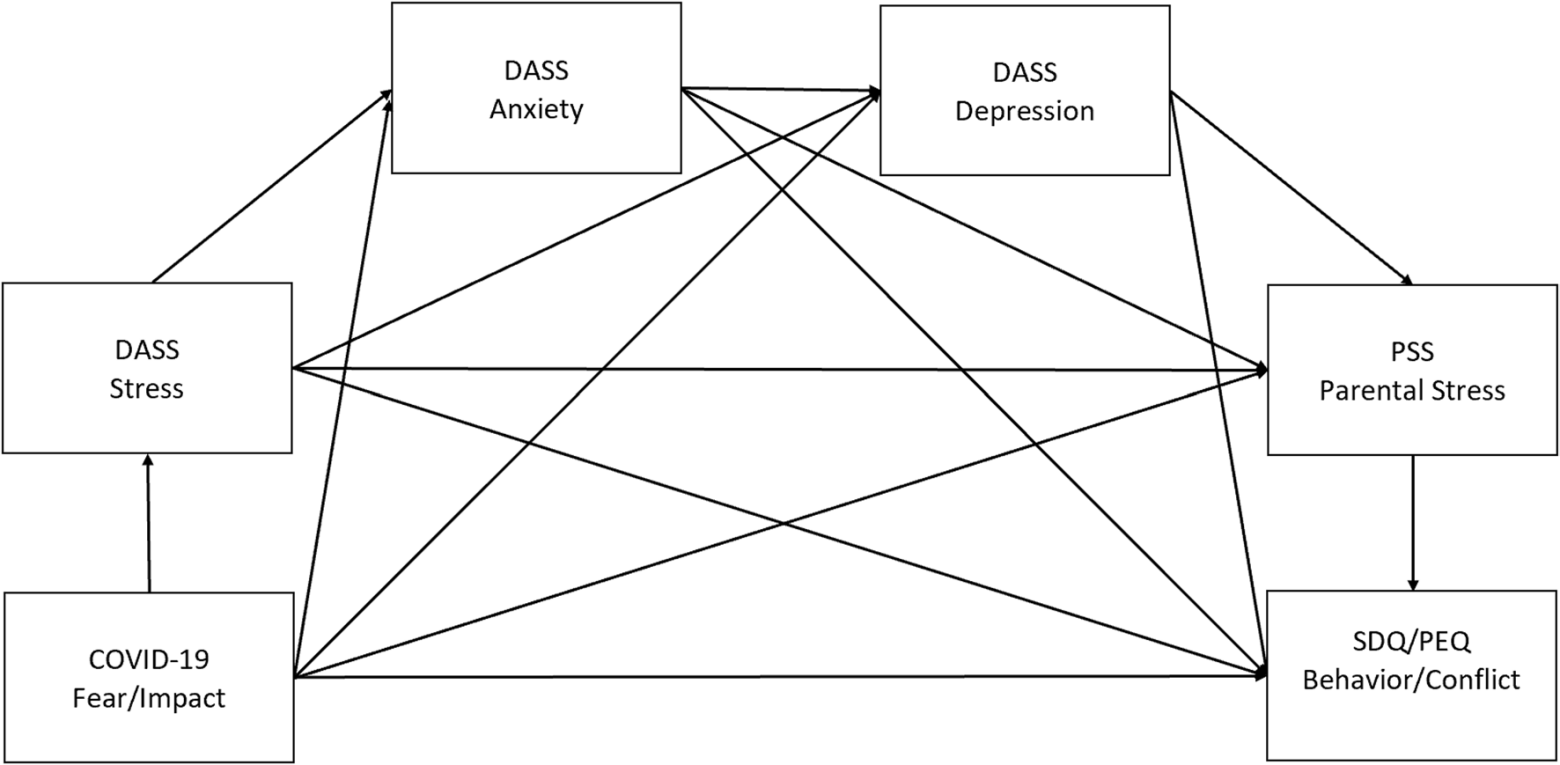
Full mediation models with two predictors (COVID-19 fear, COVID-19 impact) and four outcomes (SDQ, PEQ).

See Fig. 2 and Table 4 for the mediation model and a list of all indirect pathways. The total effect of COVID-19 Fear on the total SDQ score, like its direct and total indirect effects, were never different from zero. On the other hand, the total effect of the COVID-19 Impact on the total SDQ score was significantly different from zero, as was its total indirect effect. However, the direct effect of COVID-19 Impact on total SDQ was not significant. The mediation was thus complete for the COVID-19 impact.

**Table 4:** List of indirect pathways for the mediation models.

Indirect pathway number	First variable	Second variable	Third variable	Fourth variable	Fifth variable	Sixth variable
1	COVID-19	DASS stress	SDQ/PEQ			
2	COVID-19	DASS anxiety	SDQ/PEQ			
3	COVID-19	DASS depression	SDQ/PEQ			
4	COVID-19	Parental stress	SDQ/PEQ			
5	COVID-19	DASS stress	DASS anxiety	SDQ/PEQ		
6	COVID-19	DASS stress	DASS depression	SDQ/PEQ		
7	COVID-19	DASS stress	Parental stress	SDQ/PEQ		
8	COVID-19	DASS anxiety	DASS depression	SDQ/PEQ		
9	COVID-19	DASS anxiety	Parental stress	SDQ/PEQ		
10	COVID-19	DASS depression	Parental stress	SDQ/PEQ		
11	COVID-19	DASS stress	DASS anxiety	DASS depression	SDQ/PEQ	
12	COVID-19	DASS stress	DASS anxiety	Parental stress	SDQ/PEQ	
13	COVID-19	DASS stress	DASS depression	Parental stress	SDQ/PEQ	
14	COVID-19	DASS anxiety	DASS depression	Parental stress	SDQ/PEQ	
15	COVID-19	DASS stress	DASS anxiety	DASS depression	Parental stress	SDQ/PEQ

DASS: Depression Anxiety Stress Scales, PSS: Parental Stress Scale, SDQ: Strengths and Difficulties Questionnaire, PEQ: Parental Environment Questionnaire (Parent–Child Conflict subscale).

**Figure 3:**
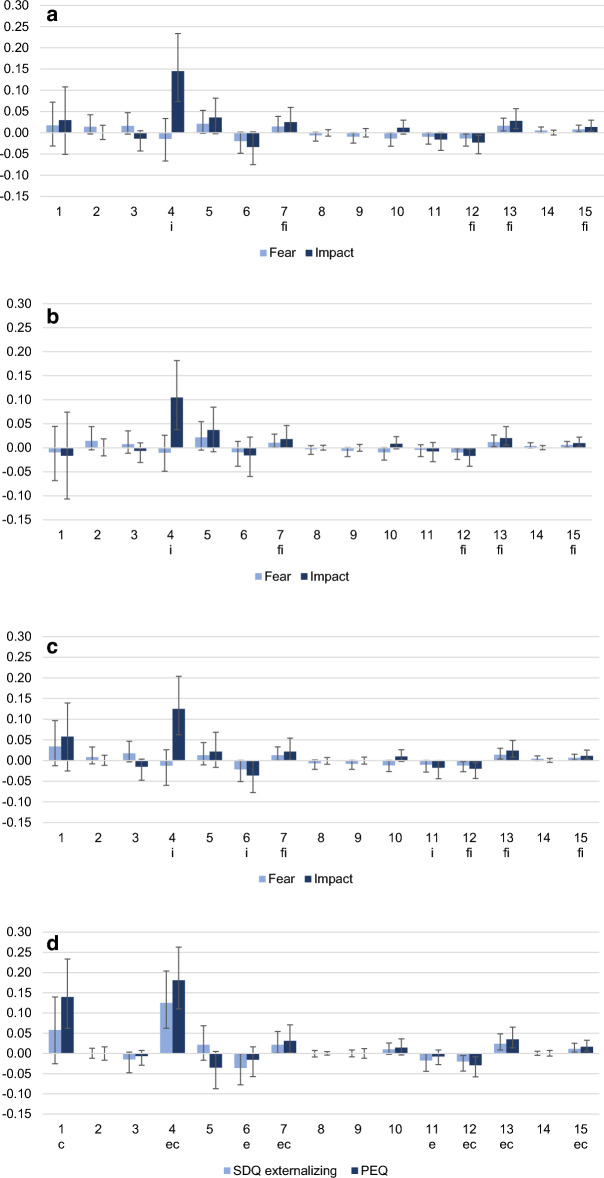
(**a**) Standardized coefficients for indirect pathways from COVID-19 fear or impact to SDQ total scores. Error bars indicate bootstrap lower and upper limit of 95% confidence intervals. f and i indicate coefficients with coefficient not including, and thus different from, zero. (**b**) Standardized coefficients for indirect pathways from COVID-19 fear or impact to SDQ internalizing scores. Error bars indicate bootstrap lower and upper limit of 95% confidence intervals. f and i indicate coefficients with coefficient not including, and thus different from, zero. (**c**) Standardized coefficients for indirect pathways from COVID-19 fear or impact to SDQ externalizing scores. Error bars indicate bootstrap lower and upper limit of 95% confidence intervals. f and i indicate coefficients with coefficient not including, and thus different from, zero. (**d**) Standardized coefficients for indirect pathways from COVID-19 impact to SDQ externalizing or PEQ parent–child conflict scores. error bars indicate bootstrap lower and upper limit of 95% confidence intervals. e (externalizing) and c (conflicts) indicate coefficients with coefficient not including, and thus different from, zero.

Standardized path coefficients for the full mediation model are presented in Fig. 3a. Five indirect paths (among 15 possible paths) mediated the effects of the COVID-19 Fear and Impact on the total SDQ scores. Four indirect paths (#7, 12, 13, 15) were common to both models; they all included the effect of COVID-19 Fear or Impact on DASS stress as the first step, and the effect of parental stress on child behavioural difficulties as the final step. The last indirect path (#4) was only significant in the model for the COVID-19 Impact, where parental stress was a unique mediator for the child behavioural difficulties. This path was significantly stronger than any other indirect path and accounted for 47.34% of the total indirect effect of the COVID-19 Impact on the SDQ total score. Thus, the parents who reported a greater impact of COVID-19 reported more parental stress and ultimately more behavioural problems in their child.

Among the covariables, the time elapsed since the emergency declaration was associated with an increase in parents’ DASS stress scores (*b* = 0.02, *p* = 0.04). Lower income was associated with an increase in parents’ DASS anxiety scores (*b* = − 0.53, *p* = 0.0002).

#### SDQ internalizing and externalizing scores

Using the same mediation model, we found similar results when we replaced the SDQ total score with the SDQ internalizing and externalizing scores. Again, it was not the fear of COVID-19, but its impact that significantly affected the SDQ internalizing and externalizing scores. The total effect of COVID-19 Fear on the internalizing or externalizing SDQ scores, like its direct and total indirect effects, were never different from zero. In contrast, the total effect and indirect effects of COVID-19 Impact on internalizing or externalizing SDQ scores were always significantly different from zero, but not its direct effect, which reflected complete mediation. The same five indirect paths (Fig. 3b and c) that mediated the SDQ total score also mediated the effect of COVID-19 Impact on SDQ internalizing and externalizing scores. Four indirect pathways (#7, 12, 13, 15) included the effect of COVID-19 Impact on DASS stress as a first step, and the effect from parental stress to the internalizing/externalizing SDQ scores as the final step. The most important path (#4) had only one mediator: parental stress. In addition, for the effects of the COVID-19 impact on externalizing SDQ scores, two additional indirect pathways (#6, 11) were significant. They both had the same first step from the COVID-19 Impact to the DASS stress, but the final step was from the DASS depression to the externalizing SDQ scores, skipping parental stress. Their effects were negative, meaning that an increase in parent’s DASS depression score were linked to decreases in the child’s SDQ externalizing scores.

As with the total SDQ model, the pathways from the COVID-19 Impact to the internalizing or externalizing difficulties going only through parental stress were again stronger than any other indirect pathway. They accounted for the largest part of the total effect: 50% of the effect on internalizing scores, and 52% on externalizing scores. The parents who reported a greater impact of COVID-19 reported more parental stress and ultimately both more internalized and externalized problems in their child.

Finally, we tested the same model with the Parent–Child Conflict subscale as an outcome (see Fig. 3d). As showed in Table 3, parent–child conflict was strongly correlated with parental stress, SDQ externalizing scores, and DASS stress. In the full mediation model, the direct effect was not significant, indicating a complete mediation. We observed the same five significant indirect pathways (#4, 7, 12, 13, 15) for the externalizing scores. However, the two additional indirect pathways (#6, 11) found for the SDQ externalizing scores where the final step went directly from DASS depression to Parent–Child Conflict were no longer significant. Instead, the indirect pathway (#1) going from COVID-19 Impact through DASS stress and then to Parent–Child Conflict was significant.

In these models, in addition to the effect of the time elapsed since the emergency declaration on parent’s DASS stress scores, and of lower income on parent’s DASS anxiety scores, we found that SDQ internalizing problems were associated for children with older age (β = 0.19, *p* < 0.05), while SDQ externalizing problems were associated with younger age (β = − 0.26, *p* < 0.01) and being male (β = 1.66, *p* < 0.01). By contrast, Parent–Child Conflict tended to be only associated with older age (β = 0.32, *p* = 0.05).

#### Family functioning in a moderated mediation model

**Figure 4:**
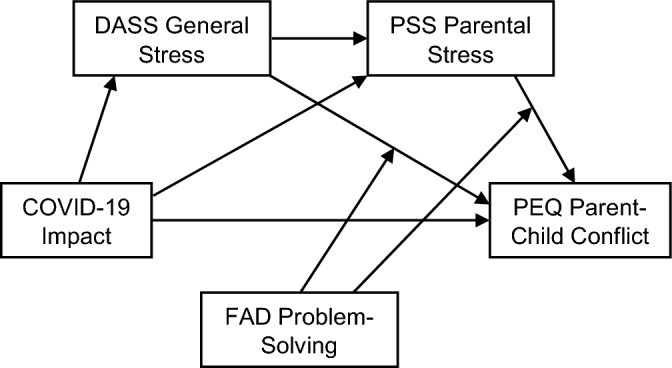
Moderated mediation model.

To test the moderated mediation model of family functioning, the impact of COVID-19 was used as the predictor and the Parent–Child Conflict subscale from the PEQ was used as the outcome (see Fig. 4). DASS stress and parental stress were retained as mediators. DASS anxiety, depression, and COVID-19 Fear were included as covariates, in addition to the same previous covariates*.*

**Figure 5:**
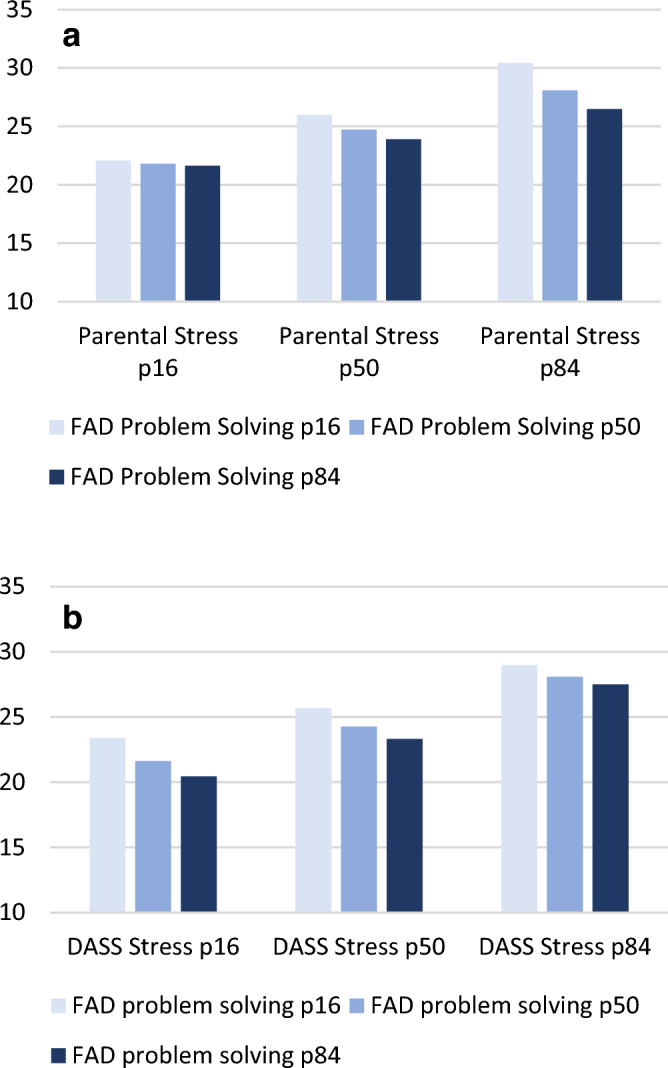
(**a**) Conditional effects of parental stress on parent–child conflict according to FAD problem solving skills scores (percentiles 16, 50 and 84). (**b**) Conditional effects of DASS stress on parent–child conflict according to FAD problem solving skills scores (percentiles 16, 50 and 84).

In this model, there are three indirect pathways from COVID-19 Impact to Parent–Child Conflict: through parental stress only, through DASS stress only, or through DASS stress and parental stress. The indirect effect of the impact of COVID-19 on parent–child conflict through parental stress only was significantly moderated (index = − 0.0950; SE = 0.0374; Boot 95% CI = − 0.1762, − 0.0279) by the family’s ability to solve problems. The impact of COVID-19 increased parental stress, which in turn increased parent–child conflict. However, the latter effect decreased with increasing abilities to resolve problems within the family. In contrast, the moderation effect of problem-solving abilities in the family was not significant when examining the indirect path through DASS stress as a mediator (index = − 0.0094; SE = 0.0118; Boot 95% CI = − 0.0112, 0.0358). When examining the indirect pathway going through both mediators, we found a small intermediate moderation effect that just failed to reach significance (index = − 0.0048; SE = 0.0039; Boot 95% CI = − 0.0148, 0.0001). Figure 5 illustrates this moderation effect. Parent–child conflict increased when parental stress or DASS stress increased. The protective effect of FAD Problem Solving skills was stronger as parental stress increased, but not as the DASS stress increased.

## Discussion

The main finding of this study is that the impact of the COVID-19 policies increased parental stress, which in turn caused poorer mental health in children, particularly for externalizing problems and parent–child conflict. In addition, we found that a family resiliency factor, the ability to solve problems within the family, mitigates the negative effects of parental stress on parent–child conflict specifically. Before drawing practical conclusions from these findings, we will comment on some general aspects of the results.

The main cause of the deterioration in children's mental health was the difficulty in coping with COVID-19 policies, but not the fear of COVID-19 itself. However, the fear of COVID-19 itself impacted parents’ mental health: fear was associated with increased general stress, worsened anxiety and depression in parents. While fear of COVID-19 did have an effect on parental mental health, this effect did not translate into worsened child mental health and parent–child conflict primarily because fear of COVID-19, unlike the impact of COVID-19, had only a minor effect on parental stress. Parental stress, especially for mothers who accounted for 80% of the respondents was the main mediator of the effect of COVID-19. Therefore, any deterioration in parental mental health only had a negative effect on children's mental health if specific mediators were affected, such as parental stress. This implies that when designing interventions, the first step is to clearly define the specific mediators to target, based on the primary outcome.

Parental stress was a systematic mediator in the significant pathways from COVID-19 impact to child behavioural difficulties. Also, when more than one mediator was involved, the first step always was general stress as measured by the DASS. When DASS anxiety and depression were part of the pathway, the indirect effects of the pathways were reduced and sometimes even reversed, but still small. This observation is consistent with the results obtained in the general population. In Singapore, parental stress mediated the effect from the perceived impact of COVID-19 to harsh parenting and decreased parent–child closeness^8^. Parental stress also mediated the effect from parents' perceived distress to worsening of SDQ hyperactivity/inattention, conduct, and emotional problem scores^10^. This suggests that the same universal approaches could be used for all families to mitigate the impact of the COVID-19 crisis, although they have to be adapted for families and children with severe mental health challenges.

We controlled several covariates in our analyses to evaluate the effects of the impact of COVID-19. The associations found for these covariates turned out to be consistent with the literature while clarifying some aspects of the model. For children, internalized problems were associated with older age and externalized problems with younger age, a pattern found in the general population^29^. Increased parent–child conflict for older children may be explained by the greater difficulty adolescents have in complying with parental rules and restrictions. For parents, the time since the declaration of the state of emergency was associated with an increase in general stress, but not in parental stress. The persistence of the crisis and the uncertainty about the return to normality had cumulative effects on general stress. But in the first year after March 17, 2020, Ontario experienced three periods of school closures separated by periods of reopening and the 2020 summer vacation. Parental stress is more sensitive to these alternations that are not captured by a simple time measure. Finally, parental anxiety was lower in those with higher income, reflecting a well-recognized social inequality throughout the COVID-19 crisis^30^.

But the clinically significant result is that the ability to solve problems at the family level moderates the effect of parental stress on parent–child conflict. This moderating effect is specific to parental stress and is not significant when considering the indirect route through general stress. It is significant after controlling for level of parental psychopathology, family income, time since the onset of the emergency, and children's age and gender. A preventive intervention focused on strengthening problem-solving skills at the family level could be an effective prevention strategy to limit parent–child conflict especially for high parental stress. This effect is consistent with the lower level of externalized symptoms associated with greater use of an individual-level problem-solving strategy demonstrated in a meta-analysis^31^. In addition, the relationship between parent-adolescent conflict and externalized/internalized problems in adolescents was found to be moderated by the type of conflict resolution. Adolescents with a high level of problem solving had better adjustment than those with a low level^32^.

This study has several limitations. Because mediation involves a process that takes place over time, it is best captured by longitudinal data. Examining this mediation through cross-sectional data, however, is justified in our study by the fact that these relationships are nearly simultaneous, unfolding rapidly in repeated clashes. COVID policies (e.g., closing school, working remotely) had an immediate effect on parental stress (e.g., finding a new balance between parenting and work responsibilities), which could quickly exacerbate parent–child conflict (parent's role in distance learning, restricting contact with friends). In addition, mediation on cross-sectional data requires that the temporal ordering of the variable being examined be correct. There is no doubt that the impact of COVID is a causal factor, and the objective of the research was to estimate how this impact may affect stress (specifically parental stress) and in turn child behavior (specifically parent–child conflict). In addition, in a follow-up study, we manipulated, through a randomized intervention (Collaborative Problem Solving), the effect of the mediator on the outcome, a design that could more clearly show the causal implication of the mediating pathway.

We did not stratify by diagnosis or broad categories of diagnosis for the several reasons. First, as the children were followed by different mental health professionals, a formal mental health diagnosis was not always made, and when diagnoses were available, there were often multiple. But the main reason was that our hypothesis was that parental stress was a mediator of the impact of COVID-19 in this high-risk sample, as in the general population, irrespective of diagnosis. However, it is possible that some specific mental conditions are more, or less, sensitive to the effect of parental stress.

We measured the impact of COVID-19 in parents, but not directly in children. This was not possible because of the very large age range and the clinical heterogeneity of the sample, and the fact that COVID-19 restrictions did not allow us to assess the children in person. The direct effect of the COVID-19 impact on child mental health can be understood through the modeling and reinforcement of parents’ response on their child’s behavior. However, some direct and child-specific aspects (such as limited relationships with peers and friends, or changes in mental health care) were not measured, but most likely did have an impact on their mental health.

This study also has some strengths. We examined a wide range of variables that can impact behavioural difficulties in children, including both general and parental stress in a relatively large sample aged 4 to 18 years old. Most importantly, we were able to study a very vulnerable pediatric population that suffered more from COVID-19 restrictions than the general population and was difficult to engage in research because families were overburdened. This study allowed to propose a family intervention, which we then tested in in a randomized trial by delivering a virtual, abbreviated version of the Collaborative Problem Solving^33^ approach to parenting, the results of which will be presented separately.

### Supplementary Information

Supplementary Information.

## References

[1] Conger RD, Ge X, Elder GH, Lorenz FO, Simons RL (1994). Economic stress, coercive family process, and developmental problems of adolescents. Child Dev..

[2] Masarik AS, Conger RD (2017). Stress and child development: A review of the Family Stress Model. Curr. Opin. Psychol..

[3] Cost, K. T. et al. Mostly worse, occasionally better: Impact of COVID-19 pandemic on the mental health of Canadian children and adolescents. Eur. Child. Adolesc. Psychiatry. 31(4), 671–684. 10.1007/s00787-021-01744-3 (2022). 10.1007/s00787-021-01744-3 PMC7909377 33638005

[4] Bussières, E.-L. et al. Consequences of the COVID-19 pandemic on children's mental health: A meta-analysis. Front Psychiatry. 12, 691659. 10.3389/fpsyt.2021.691659 (2021). 10.3389/fpsyt.2021.691659 PMC8672800 34925080

[5] Kelley ML, Self-Brown S, Le B, Bosson JV, Hernandez BC, Gordon AT (2010). Predicting posttraumatic stress symptoms in children following Hurricane Katrina: A prospective analysis of the effect of parental distress and parenting practices. J. Trauma. Stress.

[6] Deater-Deckard K (1998). Parenting stress and child adjustment: Some old hypotheses and new questions. Clin. Psychol. Sci. Pract..

[7] Moscardino U, Dicataldo R, Roch M, Carbone M, Mammarella IC (2021). Parental stress during COVID-19: A brief report on the role of distance education and family resources in an Italian sample. Curr. Psychol..

[8] Chung G, Lanier P, Wong PYJ (2020). Mediating effects of parental stress on harsh parenting and parent–child relationship during coronavirus (COVID-19) pandemic in Singapore. J. Fam. Violence.

[9] Spinelli M, Lionetti F, Pastore M, Fasolo M (2020). Parents' stress and children's psychological problems in families facing the COVID-19 outbreak in Italy. Front. Psychol..

[10] Romero E, López-Romero L, Domínguez-Álvarez B, Villar P, Gómez-Fraguela JA (2020). Testing the effects of COVID-19 confinement in Spanish children: The role of parents’ distress, emotional problems and specific parenting. Int. J. Environ. Res. Public Health.

[11] Walsh F (2003). Family resilience: A framework for clinical practice. Fam. Process.

[12] Graziano PA, McNamara JP, Geffken GR, Reid A (2011). Severity of children's ADHD symptoms and parenting stress: A multiple mediation model of self-regulation. J. Abnorm. Child Psychol..

[13] Theule J, Wiener J, Tannock R, Jenkins JM (2013). Parenting stress in families of children with ADHD: A meta-analysis. J. Emot. Behav. Disord..

[14] Amirova A, CohenMiller A, Sandygulova A (2022). The effects of the COVID-19 pandemic on the well-being of children with autism spectrum disorder: Parents’ perspectives. Front. Psychiatry..

[15] Ahorsu DK, Lin C-Y, Imani V, Saffari M, Griffiths MD, Pakpour AH (2020). The fear of COVID-19 scale: Development and initial validation. Int. J. Ment. Heal. Addict..

[16] Lovibond PF, Lovibond SH (1995). The structure of negative emotional states: Comparison of the Depression Anxiety Stress Scales (DASS) with the Beck Depression and Anxiety Inventories. Behav. Res. Ther..

[17] Crawford JR, Henry JD (2003). The Depression Anxiety Stress Scales (DASS): Normative data and latent structure in a large non-clinical sample. Br. J. Clin. Psychol..

[18] Berry JO, Jones WH (1995). The parental stress scale: Initial psychometric evidence. J. Soc. Pers. Relat..

[19] Epstein NB, Baldwin LM, Bishop DS (1983). The McMaster family assessment device. J. Marital Fam. Ther..

[20] Goodman R (1997). The strengths and difficulties questionnaire: A research note. J. Child Psychol. Psychiatry.

[21] Goodman R (1999). The extended version of the Strengths and Difficulties Questionnaire as a guide to child psychiatric caseness and consequent burden. J. Child Psychol. Psychiatry.

[22] Bourdon KH, Goodman R, Rae DS, Simpson G, Koretz DS (2005). The strengths and difficulties questionnaire: U.S. normative data and psychometric properties. J. Am. Acad. Child Adolesc. Psychiatry.

[23] Goodman R (2001). Psychometric properties of the strengths and difficulties questionnaire. J. Am. Acad. Child Adolesc. Psychiatry.

[24] Miller PA, Hauser R, Grotevant HD, Carlson CI (1989). Self-report measures of parent-child relationships. Family Assessment: A Guide to Methods and Measures.

[25] Moos, R. H. & Moos, R. S. (eds.) Family Environment Scale manual 2nd ed. (Consulting Psychologists Press, Palo Alto, CA, 1986).

[26] Burt SA, McGue M, Iacono W, Krueger R (2006). Differential parent-child relationships and adolescent externalizing symptoms: Cross-lagged analyses within a monozygotic twin differences design. Dev. Psychol..

[27] Hayes AF (2013). Introduction to Mediation, Moderation, and Conditional Process Analysis: Methodology in the Social Sciences.

[28] Shaffer, D. et al. A children's global assessment scale (CGAS). Arch. Gen. Psychiatry. 40(11), 1228–1231 (1983). 10.1001/archpsyc.1983.01790100074010 6639293

[29] Achenbach TM, Howell CT, Quay HC, Conners CK, Bates JE (1991). National survey of problems and competencies among four- to sixteen-year-olds: Parents' reports for normative and clinical samples. Monogr. Soc. Res. Child Dev..

[30] Hall LR, Sanchez K, da Graca B, Bennett MM, Powers M, Warren AM (2021). Income differences and COVID-19: Impact on daily life and mental health. Popul. Health Manag..

[31] Compas BE, Jaser SS, Bettis AH, Watson KH, Gruhn MA, Dunbar JP, Williams E, Thigpen JC (2017). Coping, emotion regulation, and psychopathology in childhood and adolescence: A meta-analysis and narrative review. Psychol. Bull..

[32] Branje SJT, van Doorn M, van der Valk I, Meeus W (2009). Parent–adolescent conflicts, conflict resolution types, and adolescent adjustment. J. Appl. Dev. Psychol..

[33] Greene RW, Ablon JS, Goring JC (2003). A transactional model of oppositional behavior: Underpinnings of the Collaborative Problem Solving approach. J. Psychosom. Res..

