# Supplementary material for: Mediators and moderators of the effects of the COVID-19 crisis on parent–child conflict in children in tertiary mental health care
Source: Sci Rep. 2023 Dec 16;13:22422. doi: 10.1038/s41598-023-49409-2 (PMC10725487; doi:10.1038/s41598-023-49409-2)

**Appendix A:** Comparison of study variables for participants with full survey data (completers) and partial survey data (non-completers)

| **Variable** | **Completers**  **M (SD)** | **Non-completers**  **M (SD)** | **Significance** |
| --- | --- | --- | --- |
| Child age | 13.51 (3.23) | 12.96 (3.72) | *p* > .05 |
| COVID Fear | 17.08 (5.20) | 18.00 (6.20) | *p* > .05 |
| COVID Impact | 54.54 (10.30) | 54.31 (13.72) | *p* > .05 |
| PSS Parental Stress | 47.04 (11.27) | 45.19 (11.33) | *p* > .05 |
| DASS Stress | 13.92 (9.74) | 14.47 (9.94) | *p* > .05 |
| DASS Anxiety | 6.18 (7.19) | 7.74 (9.74) | *p* > .05 |
| DASS Depression | 10.21 (10.16) | 10.72 (9.96) | *p* > .05 |
| FAD Problem Solving | 2.83 (0.48) | 2.91 (0.43) | *p* > .05 |

Note: PSS: Parental Stress Scale, DASS: Depression Anxiety Stress Scales, FAD: Family Assessment Device.

**Appendix B:** Factor matrix for the final 13 items of the COVID-19 Impact scale

| **Item number** | **Item** | **Factor score** |
| --- | --- | --- |
| 1 | I have difficulty setting up a routine at home | 0.712 |
| 2 | I have difficulty keeping up with my child’s schoolwork | 0.674 |
| 3 | It is difficult for me to do more cleaning at home | 0.661 |
| 4 | Homeschooling my child while working at home is difficult for me | 0.626 |
| 5 | It is difficult for me to do more cooking at home | 0.583 |
| 6 | My child lost some of his/her usual care | 0.545 |
| 7 | The quality of the care for my child has decreased | 0.512 |
| 8 | I had to stop or change my physical leisure activities (jogging, gym), and it is difficult for me | 0.482 |
| 9 | Changing my way of working (from home, etc.) is difficult | 0.481 |
| 10 | I had to stop or change my non-physical leisure activities (movies, restaurants, etc.), and it is difficult for me | 0.465 |
| 11 | I have difficulty using programs or websites that I need for work/shopping/schoolwork | 0.455 |
| 12 | I have difficulty with poor internet connection and computer/phone | 0.410 |
| 13 | I have difficulty staying inside most of the time | 0.406 |

Appendix C: DSQ score distribution.


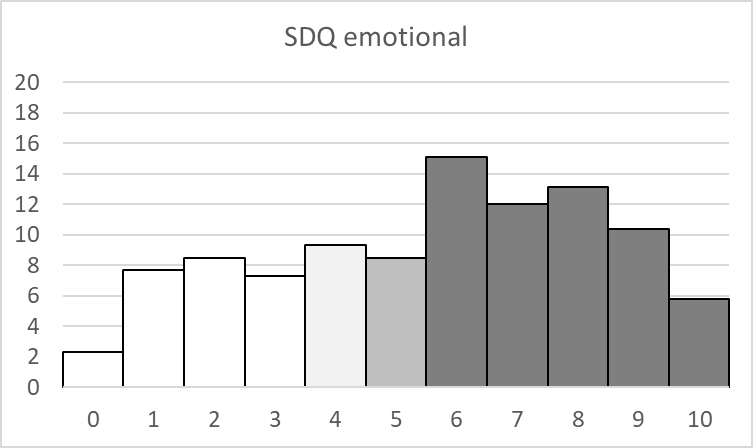


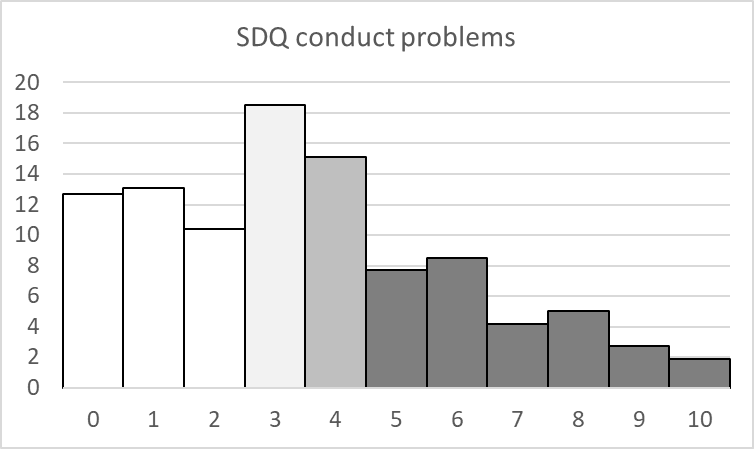


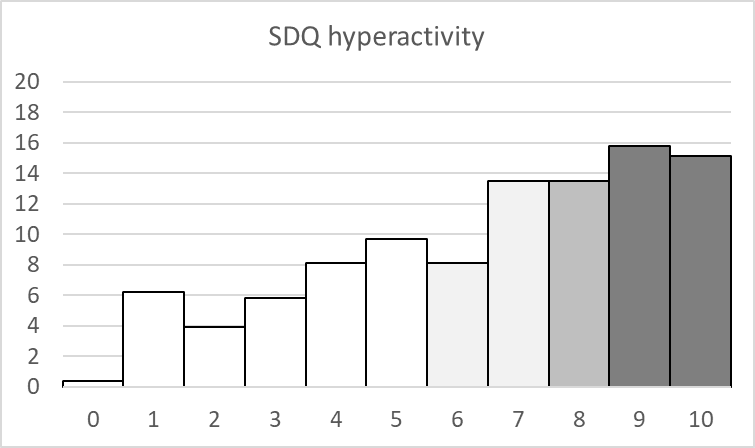


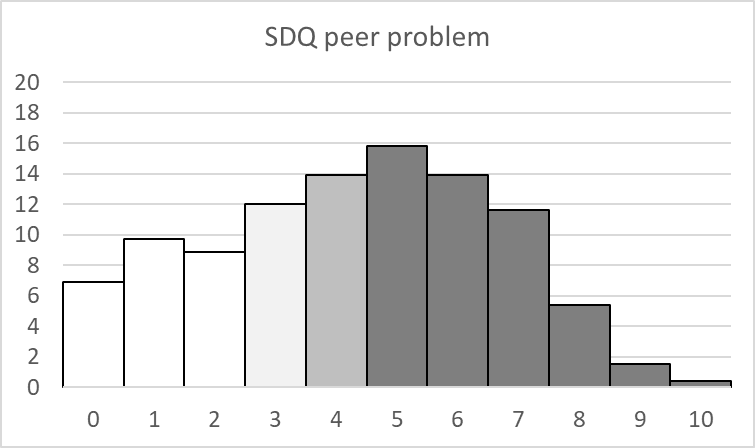


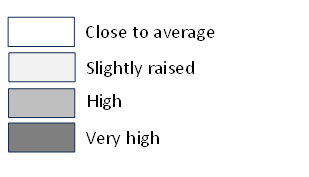


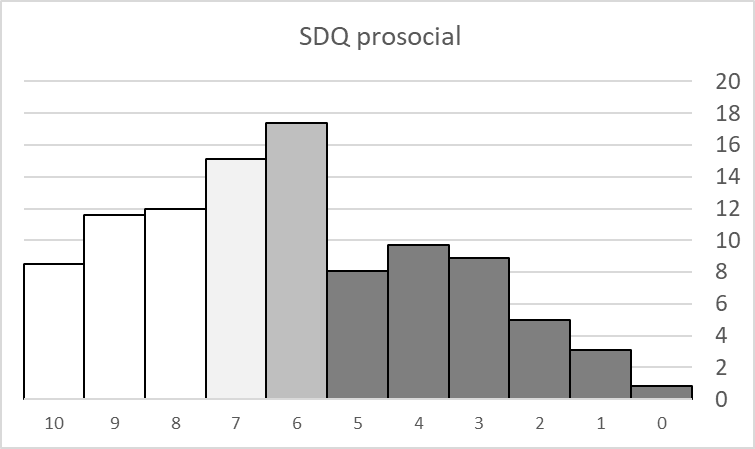


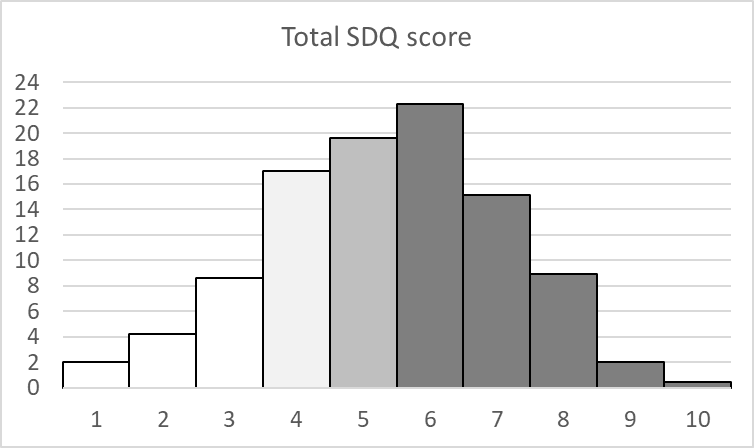


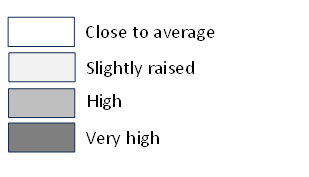

Supplement: Supplementary file 1 — Supplementary Information. [file 41598_2023_49409_MOESM1_ESM.docx]
